# Immature Neurons in the Postnatal Brain: Markers, Modulation, and Involvement in Normal and Aberrant Plasticity

**DOI:** 10.3390/ijms27156696

**Published:** 2026-07-27

**Authors:** Viacheslav Riga, Victor Aniol, Natalia Gulyaeva

**Affiliations:** Department of Functional Biochemistry of the Nervous System, Institute of Higher Nervous Activity and Neurophysiology, RAS, Butlerov Street 5A, Moscow 117485, Russia; slavariga@bk.ru (V.R.); aniviktor@yandex.ru (V.A.)

**Keywords:** neuronal development, neurogenesis, piriform cortex, neocortex, evolution, neuronal precursors, epilepsy, DCX, PSA-NCAM, structural plasticity

## Abstract

Cortical immature neurons (cINs) represent a unique population of prenatally generated, non-dividing neurons that maintain an immature phenotype, characterized by doublecortin (DCX) and polysialylated neural cell adhesion molecule (PSA-NCAM) expression, into adulthood. Unlike canonical adult neurogenesis involving continuous neuron generation from stem cell niches, cINs constitute a distinct form of structural plasticity termed “neurogenesis without division”. This review comprehensively examines the molecular markers, morphological diversity, developmental origins, and maturation trajectories of cINs across species. We highlight the striking inverse interspecies relationship between cIN abundance and canonical adult neurogenesis, reflecting distinct biophysical and structural shifts in neural plasticity mechanisms across mammalian lineages. Furthermore, we discuss factors modulating cIN phenotype, including neurotransmitter systems, stress, sensory experience, and aging. Clinical evidence implicating cIN alterations in temporal lobe epilepsy, traumatic brain injury, and stroke is evaluated, revealing potential roles in both pathological circuit remodeling and endogenous repair. Critical gaps remain regarding the molecular programs maintaining immaturity, differentiation triggers, and the functional consequences of circuit integration. Understanding cIN biology offers new perspectives on cortical plasticity and may inform therapeutic strategies targeting endogenous cellular reserves for brain repair.

## 1. Introduction

Adult neurogenesis represents possibly one of the most intriguing forms of neuroplasticity in the postnatal mammalian brain and, in this sense, it has attracted the close attention of researchers around the world over the past few decades. The pioneering studies of Altman and Das, who initially showed the generation of new neurons in the adult mammalian brain by using a 3H-thymidine method, date back to the 1960s, but at that time they were generally left without proper attention [1,2,3]. True progress in this field started again in the 1990s with the identification of numerous molecular markers typical of immature neurons and the development of immunohistochemical methods to detect them. In combination with synthetic nucleotide labeling (primarily 5-bromo-2′-deoxyuridine), this gave researchers a range of powerful instruments for detailed investigation of adult neurogenesis. To date, the total number of publications on the topic of adult neurogenesis exceeds 14,000.

Attempts have also been made to understand the significance of this form of plasticity and the role of young cells in the functioning of the adult brain under normal and pathological conditions. The first straightforward assumptions regarding the reparative function of newly born neurons were rejected [4] shortly after the strict spatial localization and quantitative limitations of this process became clear. Along with this, experimental findings gradually accumulated, indicating changes in the rate of adult neurogenesis. Acute and chronic stress, social isolation, aging, exposure to corticosteroids, and neuroinflammation were accompanied by decreased generation of new neurons. Increased neurogenesis was observed after exposure to enriched-environment conditions and physical exercise during pregnancy in females [5,6,7]. Some pathological situations were also found to be accompanied by enhanced neurogenesis: seizures of different origin [8], ischemic stroke [9] or brain injury [10]. Based on the accumulated experimental material, several intriguing biomedical hypotheses have been put forward linking changes in the normal course of hippocampal neurogenesis and the development of various neurological and psychiatric pathologies. For example, the discovery of increased formation and atypical maturation of new cells after severe seizures formed the basis for the hypothesis of the involvement of neurogenesis in the development of epilepsy [11]. Indeed, after seizures, resting progenitor cells of neurons are usually involved in the cell cycle, after which their descendants can migrate to the polymorphic layer of the dentate gyrus instead of the granular one, as well as give off atypical apical dendrites and demonstrate increased branching of regrowing axons (so-called “sprouting of mossy fibers”). The collaterals of these axons can be directed into the dentate gyrus instead of the CA3 field, thus creating opportunities for local circulation of excitation. Similar structural changes have also been observed in human epileptic tissues, obtained either post-mortem or following amygdalohippocampectomy for pharmacoresistant temporal lobe epilepsy. This parallelism strongly supports the hypothesis that young neurons, generated during seizure-induced neurogenesis, actively participate in forming the epileptogenic focus. An additional argument in favor of such involvement was also the fact that young neurons tend to be more excitable for stimulation.

Another interesting hypothesis was the possible role of neurogenesis in the development of depression [12]. By that time, it was known that chronic stress and increased glucocorticoid level, which play a role in the development of depressive disorders, may reduce the proliferation and survival of newly born cells in the hippocampus [13]; subsequently confirmed by [14]. On the other hand, treatment with antidepressant drugs has been found to enhance cell proliferation in the hippocampus in laboratory animals [15,16,17]. The effect of antidepressant drugs usually takes several weeks to completely develop, corresponding to the time required for neuronal differentiation of newly born cells and their functional integration into the hippocampal neuronal circuits. In addition, experimental conditions such as electroconvulsive shock, physical activity, and environmental enrichment, on the one hand, lead to an increase in adult neurogenesis, and on the other to a decrease in depressive-like symptoms in experiments [18,19]. Based on these consistent data, it was hypothesized that development of depressive-like symptoms may involve decreased hippocampal neurogenesis, and the therapeutic effect of antidepressant drugs is associated with their ability to restore its normal level [12].

However, despite the huge amount of data obtained in animal experiments, their possible translation to humans is essentially compromised by the lack of a complete consensus on the prominence of adult hippocampal neurogenesis in the human brain [20]. In addition, it became clear that some species and probably even orders of the mammalian class (like cetaceans) demonstrate very low levels of adult hippocampal neurogenesis [21]. Thus, there is reason to suppose that adult hippocampal neurogenesis is far from being a common mechanism of neuroplasticity in all mammals, which casts doubt on the simple translation of laboratory findings regarding adult hippocampal neurogenesis to our understanding of the mechanisms of human brain plasticity.

On the other hand, recent studies have shown that large-brained mammals such as primates (and humans) have large numbers of neurons continuously expressing typical markers of neurogenesis in the absence of signs of cell proliferation and birth. It was found that populations of neurons located in the second layer of the cerebral cortex, formed in utero and presumably not dividing, continue to express molecules typical of immature neurons throughout life. These cells can retain the potential to differentiate into mature neurons capable of integrating into existing neural circuits. According to the current hypothesis, these populations of immature neurons may represent a reservoir of plastic cells for those mammalian species that are not characterized by pronounced neurogenesis in adulthood [22]. The issue of cortical immature neurons (cINs), along with the concept of “neurogenesis without division”, is relatively new and still not fully understood [23,24,25]. While “canonical” neurogenic regions in the subventricular zone and the dentate gyrus are characterized by ongoing generation of new cells by mitosis and their subsequent immediate differentiation into mature cells (mainly neurons), the cINs in the piriform cortex exist in an undifferentiated form, retaining their ability to completely differentiate for long periods of time. The main differences between adult-born neurons in “canonical” neurogenic areas and cINs in the piriform cortex are listed in Table 1. The molecular and cellular mechanisms that allow these neurons to suspend their maturation and subsequently reactivate it in adulthood are still unknown. In addition, their prevalence in the brain has not yet been fully studied [26,27], and it remains unclear whether they can be activated in response to injury, inflammation, or neurological pathologies. Here, we will try to give a brief overview of up-to-date information concerning the cellular and molecular characteristics of cINs, the regulatory mechanisms of their maturation, and their involvement in brain functioning under normal and pathological conditions.

## 2. Identification, Representation and Functional Significance of cINs

### 2.1. cIN Markers

The molecular characterization of cINs relies on a complex array of markers that collectively define their immature state while distinguishing them from other cell populations. Understanding the limitations and specificities of these markers is crucial for accurate identification and functional interpretation (Table 2).

Cytoskeletal and migration markers form the primary identification toolkit for cINs. Doublecortin (DCX) is a microtubule-associated protein essential for cortical development, which binds to microtubules through its tandem doublecortin-like domains and regulates neuronal migration and dendrite growth during brain development [62,63]. DCX stabilizes and nucleates microtubules, preferentially promoting formation of 13-protofilament microtubules, and mutations in the DCX gene cause neuronal migration disorders such as X-linked lissencephaly, characterized by intellectual disability and epilepsy [62]. DCX shows strong expression in cINs [31,36,40,64,65,66] but is not exclusive to these cells, also appearing in adult-born neurons within neurogenic niches and some mature interneuron populations [23,67,68]. The polysialylated form of neural cell adhesion molecule (PSA-NCAM) is a post-translationally modified glycoprotein in which linear chains of α2,8-linked sialic acid polymers are added to the N-glycans of NCAM, creating a large negatively charged hydrated structure that reduces cell–cell adhesion [23,69,70]. PSA-NCAM plays critical role in neuronal migration, axonal growth and synaptic plasticity by increasing the lateral diffusion of NCAM in the cell membrane and modulating cell surface interactions [23,71]. Its anti-adhesive properties likely contribute to the isolation of cINs from mature circuits [35,69,72,73]. Besides cINs, PSA-NCAM is expressed by newly generated neuroblasts in the SVZ and SGZ [23,74], olfactory bulb [75] and reactive astrocytes [76]. The coexpression of DCX and PSA-NCAM in the mammalian neocortex varies from approximately 53% to over 98% [36,66]. The co-expression of DCX and PSA-NCAM confers greater specificity for identifying immature neurons than either marker alone, as demonstrated by substantially reduced NeuN co-expression in DCX+/PSA-NCAM+ cells compared to cells expressing only one marker [36]. Maturation markers provide critical information about differentiation state. NeuN expression is used as a marker specifically to detect post-mitotic neurons and is typically absent or very low in immature cINs but increases during maturation. In the rat, mouse, cat paleocortex and human neocortex most PSA-NCAM+ and DCX+-expressing cells in layer II rarely express NeuN, while complex cells (more advanced in maturation) show increasing NeuN co-expression that reaches approximately 90% in fully mature neurons [31,36,37,40,77,78,79]. Marker expression profiles of cortical immature neurons are summarized in Table 2 and will be elaborated upon in subsequent sections.

**Table 2:** The markers of cINs.

Marker	Function/Annotation	Expression in cINs	Co-Expression with DCX	Species/Area	References
DCX	Microtubule-associated migration/plasticity	Positive	100%	Neocortex (large-brain mammalian); paleocortex (rodents)	[35,45,58,80,81,82]
PSA-NCAM	Anti-adhesive molecule; synaptic insulation; delayed maturation	Positive	94%	Human temporal cortex	[83]
		Positive	98%	Human temporal cortex (TLE)	[83]
		Positive	53%	Human neocortex	[36]
		Positive	60%	Mice piriform cortex	[32]
		Positive	–	Cat neocortex	[84]
		Positive	100%	Guinea pig neocortex	[65]
NeuN	RNA-binding protein regulating neuronal differentiation and splicing/marker of mature neurons	Low	9%	Human neocortex	[36]
		Low	–	Human temporal cortex (TLE)	[58]
		Emerges with maturation	–	Mice piriform cortex	[73]
		Low	19%	Mice piriform cortex	[32]
		Low	20% of PSA-NCAM cells	Cat cerebral cortex	[84]
		Low	14% of PSA-NCAM cells	Rat piriform cortex	[31]
		Appears in type 2 cINs	–	Guinea pig neocortex	[65]
		Emerges with maturation	–	Cat neocortex	[66]
		Low	17–18%	Rodents, cat and marmoset neocortex	[33]
MAP2	Cytoskeletal protein playing a crucial role in dendritic development	Negative/low		Rat piriform cortex	[31]
Ankyrin-G	Formation and maintenance of the axon initial segment; Marker of neuronal maturation	Negative	–	Human neocortex	[36]
Reelin	Coordination of neuron migration	Negative	–	Rodent piriform cortex; guinea pig neocortex	[65,85]
c-Fos	Immediate early gene	Negative	–	Rat piriform cortex	[31]
Arc	Immediate early gene	Negative	–	Rat piriform cortex	[31]
PV	Calcium-binding proteins	Negative	–	Human/cat/guinea pig neocortex	[65,66,83,84]
		Positive	–	Cat cerebral cortex layer II	[66]
Calretinin	Calcium-binding proteins	Negative	–	Human/cat/guinea pig neocortex	[65,66,83,84]
Calbindin	Calcium-binding proteins	Negative	–	Human/cat/guinea pig neocortex	[65,66,83,84]
		Positive	–	Cat neocortex	[66]
VGLUT1	Excitatory neuron marker	Emerges with maturation	–	Human neocortex	[36]
CUX1	Excitatory neuron marker	Positive	73%	Human neocortex	[36]
CTIP2	Excitatory neuron marker	Positive	21%	Human neocortex layer II	[36]
TBR1	Postmitotic excitatory neuron marker	Positive	100%	Human neocortex	[36]
		Positive	96% of PSA-NCAM cells	Cat neocortex	[84]
		Positive	–	Murine piriform cortex	[37]
		Positive	–	Rodent piriform cortex; guinea pig and rabbit neocortex	[35]
GluN1	Subunit of NMDA receptor; marker for glutamatergic signaling	Appears in type 2 of cINs	–	Human neocortex	[36]
		Appears in type 2 of cINs	92% of PSA-NCAM cells	Rat piriform cortex	[31]
Ng	Marker of cerebral principal neurons	Negative	–	Guinea pig neocortex	[65]
CAMKII	Marker of mature principal neurons	Negative	–	Cat neocortex; rat piriform cortex	[31,84]
		Positive	2–12%	Rat piriform cortex	[37]
GR	Indicator of stress hormone signaling	Negative	–	Rat piriform cortex	[31]
NPY	Marker of the corresponding population of interneurons	Negative	–	Cat neocortex; rat piriform cortex	[31,84]
CCK	Marker of the corresponding population of interneurons	Negative	–	Cat cerebral cortex layer II; rat piriform cortex layer II	[31,84]
SRIF	Marker of the corresponding population of interneurons	Negative	–	Cat neocortex; rat piriform cortex	[31,84]
VIP	Marker of the corresponding population of interneurons	Negative	–	Cat neocortex; rat piriform cortex	[31,84]
GAD67	A key enzyme responsible for synthesizing GABA; marker of inhibitory interneurons	Negative	–	Human neocortex	[36]
		Negative	–	Cat neocortex	[84]
		Negative	–	Rat piriform cortex	[31]
		Positive	–	Guinea pig neocortex	[86]
		Emerges with maturation	–	Cat neocortex	[66]
		Low	–	Guinea pig neocortex	[65]
GABA	Marker of GABAergic neurons	Low	–	Guinea pig neocortex	[65]
		Positive	–	Rhesus monkeys	[46]
		Emerges with maturation	–	Cat neocortex	[66]
SOX2	Stem/progenitor marker	Low	–	Human temporal cortex (TLE)	[58]
CD68	Microglia marker	Positive	–	Human temporal cortex (TLE)	[58]
CD34	Marker for hematopoietic stem and progenitor cells	Negative	–	Human temporal cortex (TLE)	[58]
GFAP	Marker for astrocytes	Very low	2.7%	Human neocortex	[36]
		Negative	–	Guinea pig neocortex	[65]
		Positive	26%	Non-human primate (*Chlorocebus*) neocortex	[81]
		Negative	–	Human temporal cortex (TLE)	[58]
		Negative	–	Rat piriform cortex	[31]
PDGFRβ	A receptor of tyrosine kinase involved in cell proliferation, migration, differentiation, and survival	Positive	–	Human temporal cortex (TLE)	[58]
OLIG2	Marker for oligodendrocyte precursors and motor neuron progenitors	Low	–	Human white matter of temporal cortex (TLE)	[58]
IBA1	Marker for microglia and macrophages	Negative	–	Human neocortex layer II	[36]
		Positive	–	Human temporal cortex layer II (TLE)	[58]
Nestin	Marker for neural stem and progenitor cells	Negative	–	Human temporal cortex layer II (TLE)	[58]
		Positive	–	Rat piriform cortex	[87]
BrdU	Cell birth dating method; proliferative marker	Negative at adult labeling	–	Cat/guinea pig neocortex; rodent piriform cortex	[31,32,33,84,87,88]
		Positive	–	Rhesus monkey temporal cortex	[89]
		Positive	–	Guinea pig neocortex	[86]
Tuj1	Marker of immature neurons	Positive	43%	Human temporal cortex	[83]
		Positive	82%	Human temporal cortex (TLE)	[83]
		Positive	70%	Human neocortex (stroke)	[90]
		Positive	–	Guinea pig neocortex	[86]
CNGA-3	Marker for cells of the rostral migratory stream	Positive	98% of PSA-NCAM cells	Rat piriform cortex	[31]
p-CREB	Participates in the survival, differentiation, and maturation of newborn neurons	Positive	95% of PSA-NCAM cells	Rat piriform cortex	[31]
ßIV-spectrin	Marker for axon initial segments	Positive	–	Mice piriform cortex	[79]
Caspase 3	Caspase-3 orchestrates execution-phase apoptosis events	Negative	–	Guinea pig neocortex	[91]
nNOS	Marker of nitrergic neurons	Positive	–	Cat neocortex	[66]
		Low	–	Guinea pig neocortex	[65]
		Negative	–	Cat neocortex	[84]
NADPH-d	Marker of nitrergic neurons	Positive	–	Cat neocortex	[66]
		Low	–	Guinea pig neocortex	[65]
MCM2	Proliferative marker	Negative	–	Human temporal cortex (TLE)	[58]
PCNA	Proliferative marker	Positive	<6%	Human temporal cortex	[83]
		Positive	64%	Human temporal cortex (TLE)	[83]
Ki-67	Proliferative marker	Very low	–	Human neocortex	[92]
		Positive	–	Human neocortex (TBI)	[92]
		Negative	–	Human neocortex (stroke)	[90]
		Negative		Rabbit and sheep neocortex	[33]
		Positive	–	CD1 mice piriform cortex	[54]
TUC4	Marker of newborn and immature neurons	Positive	69% of PSA-NCAM cells	Rat piriform cortex	[31]
NG2	Marker for oligodendrocyte precursor cells	Negative	–	Human neocortex	[36]
		Low	–	Rat piriform cortex	[31]

MAP2—microtubule associated protein 2; NeuN—neuronal nuclear antigen; PV—parvalbumin; VGLUT1—vesicular glutamate transporter 1; CUX1—Cut-like homeobox 1; CTIP2—COUP-TF-interacting protein 2; TBR1—T-Box Brain 1; GluN1—N1 subunit of the NMDA receptor complex; GR—glucocorticoid receptor; NPY—neuropeptide Y; Ng—neurogranin; GAD67—glutamic acid decarboxylase; SOX2—SRY-box transcription factor 2; GFAP—Glial Fibrillary Acidic Protein; CAMKII—Ca^2+^/calmodulin-dependent protein kinase II; nNOS—neuronal isoform of nitric oxide synthase; NADPH-d—β-nicotinamide adenine dinucleotide phosphate diaphorase; CCK—cholecystokinin; SRIF—somatotropin release inhibiting factor; PDGFRβ—Platelet-Derived Growth Factor Receptor beta; MCM2—cell cycle marker 2; IBA1—Ionized calcium-binding adapter molecule 1; Tuj1—βIII-tubulin; PCNA—Proliferating Cell Nuclear Antigen; VIP—vaso-intestinal peptide; CNGA-3—A3 subunit of the cyclic nucleotide-gated ion channel; p-CREB—phosphorylated Cyclic AMP Response Element-Binding protein; NG2—Nerve-Glial antigen 2; TUC4—TOAD-64/Ulip/CRMP family protein; NADPH-d—NADPH diaphorase; TBI—traumatic brain injury; TLE—temporal lobe epilepsy.

### 2.2. cINs Morphology

cINs demonstrate considerable morphological diversity, which is associated with their maturation and functional integration. F. Luzzati and colleagues identified two primary categories based on soma size, dendrite complexity, and marker expression patterns [35]. Subsequently, the morphological and electrophysiological characteristics of these cells were refined [36,37,79].

Type 1 cells, also referred to as tangled or tufted cells, are small neurons with soma diameters ranging from 3 to 9 µm [35,36]. They possess only one short process, confined within cortical layer II, and represent the majority of cINs, accounting for approximately 65% of the population [36,58]. Tangled neurons display robust expression of key immature neuronal markers, including DCX and PSA-NCAM, across their somata and processes, as well as neuron-specific class III β-tubulin, which collectively define their immature phenotype (see Table 2) [31,35,36]. Notably, these cells lack expression of markers associated with neural stem cells, such as nestin, mature neuronal markers including NeuN, CaMKII, and GAD67, oligodendrocyte marker RIP, and astrocytic marker GFAP [31,32]. Electrophysiological profiling reveals that these neurons have a lower membrane capacitance in comparison to both age-matched principal neurons of the piriform cortex and type 2 cINs [79]. From a functional standpoint, these tangled cells appear quiescent, evidenced by their lack of activity-dependent expression of immediate early genes like c-Fos and Arc [85], alongside frequent somatic ensheathment by astrocytic endfeet [31], potentially contributing to their electrophysiological silence.

Type 2, or complex/semilunar/transitional cells, are larger, with soma diameters of 9 to 17 µm [35,36]. Complex cells constitute approximately 13% of cINs [35,36]. They are multipolar neurons with more developed dendritic trees, often oriented parallel to cortical layer II [36]. These cells extend a thin axon-like process and exhibit both apical and basal dendrites [36]. Although their dendritic branching is not highly elaborate, it commonly shows varicosities and small protrusions resembling short spines. They express DCX and PSA-NCAM, albeit at lower intensity than tangled cells, and frequently show weak NeuN immunoreactivity, suggesting partial functional maturation [31,32,36]. The presence of βIV-spectrin delineating the initial segments of axons further supports the emergence of neuronal polarity and axonal differentiation in this subtype [79]. Between these two categories, a morphologically intermediate group of neurons can be identified [36]. These represent about 22% of all cINs and possess soma diameters of 9–11 µm. They typically display a thin basal process and one or two short, smooth dendrites devoid of spines or protrusions [36]. Morphological analysis reveals continuous transitions among cIN types, indicating a maturation continuum [31,37]. Because these categories represent a spectrum rather than rigidly distinct populations, overlaps in soma diameter (e.g., at 9 µm) frequently occur. In such borderline cases, classification is determined not by size alone, but primarily by dendritic complexity (the number and arborization of processes) and the onset of maturation markers (such as NeuN or βIV-spectrin). Electrophysiological and morphological data from transgenic reporter mice show that single cells can progress from tangled to complex morphologies, confirming a continuous maturation from type 1 to type 2 cells and their subsequent differentiation into mature neurons [79].

### 2.3. cIN Origin

At present, multiple lines of evidence support three potential mechanisms underlying the origin of cINs: (1) the maturation of already existing, prenatally generated immature progenitors in cortical layer II without cell division; (2) the proliferation of these progenitors; and/or (3) the migration of progenitor cells from canonical neurogenic niches into the cortical layers (see graphical abstract). The first hypothesis posits that cINs are generated during embryogenesis, but their maturation process is arrested and can be reactivated throughout the entire or the majority of the animal’s lifespan [93]. Birth dating studies using BrdU administration at various developmental timepoints consistently label cIN populations, especially in the early phases of corticogenesis (particularly at embryonic day 15.5), but not during postnatal or adult periods [31,32,33,84,87,88]. In addition to the absence of DCX and BrdU colocalization in adults, several studies investigating the neocortex of humans, sheep, and rabbits report no co-expression of DCX with other proliferation markers such as PCNA, MCM2, and Ki67 [33,58,90]. Furthermore, studies in humans, rodents, rabbits, and guinea pigs have demonstrated that the majority (up to 100%) of cINs co-express the postmitotic projection neuron marker Tbr1 [35,36,37,54,84]. These findings demonstrate that the vast majority of cortical cINs are generated during a specific developmental window in embryogenesis, corresponding to the period of peak cortical neurogenesis, after which they do not proliferate. Other studies have identified reelin-expressing cells located in close proximity to cINs in cortical layer II, suggesting that these cells may contribute to the formation of a specialized microenvironment and the maintenance of the immature phenotype of cINs, analogous to the role of Cajal–Retzius cells during prenatal neurogenesis [85]. Fate-mapping experiments using DCX-CreER^T2^/Flox-EGFP and DCX-DsRed transgenic mice confirm that these cells derive from cortical progenitors [37,79]. However, these studies did not utilize markers of neuroblast proliferation, which suggests that alternative pathways for progenitor emergence cannot be discounted. The hypothesis of local origin through cell division suggests that cINs arise from proliferating progenitors within cortical regions.

Despite numerous studies employing BrdU labeling that failed to demonstrate neurogenesis as a source of cINs (Table 2), several reports have described the expression of other proliferation markers in conjunction with DCX positivity, particularly under pathological conditions. For instance, in patients with traumatic brain injury and temporal lobe epilepsy an increased number of DCX+/PCNA+ and DCX+/Ki-67+ cells was observed compared with controls, and co-expression was also detected but at a very low level (see Section 4 for details) [83,92]. Likewise, co-localization of DCX with BrdU was noted only in a few cells within the neocortex of guinea pigs and Rhesus monkeys [86,89]. The current limited evidence suggests that cINs may have a potential proliferative capacity, possibly reflecting their role in compensating for neuroplasticity in neurological conditions. Alternatively, it could be that neuronal stem cell progeny are recruited to the lesion site.

The migration hypothesis proposes that cortical cINs originate from neural stem cells in the ventricular-subventricular zone (V-SVZ) and migrate into cortical regions via the rostral migratory stream (RMS) or alternative routes. This model draws support from evidence that SVZ-derived neurogenesis continuously generates neuroblasts migrating to the olfactory bulb throughout life [43,94]. Under pathological conditions, particularly following brain injury, stroke and certain neurodegenerative disorders, some SVZ-derived neuroblasts redirect their migration toward cortical lesions in an apparent regenerative response [95,96,97,98]. Moreover, P. Bernier and colleagues demonstrated the existence of a migratory stream from the subventricular zone (SVZ) to the amygdala, piriform cortex, and inferior temporal cortex in adult squirrel monkeys (*Saimiri sciureus*) and cynomolgus monkeys (*Macaca fascicularis*) [99].

This hypothesis is further supported by the fact that DCX modulates cytoskeletal dynamics essential for cell motility, making it a recognized marker for neuroblast migration [95,100]. Based on this, one of the earliest studies of cINs by J. Nacher et al. proposed that DCX/PSA-NCAM-expressing cells in the piriform cortex represented migrating neurons, inferred from their spindle-shaped morphology with vertically oriented processes, chain-like organization, and presence in the white matter near the piriform cortex, akin to cells in the RMS [72]. Conversely, if cINs indeed migrate radially to reach their final destination in cortical layer II, as occurs following brain injury [92,101], one would expect their presence in multiple cortical layers. However, most studies report that cINs are predominantly restricted to layer II, with only occasional extension into layers I and III [65,72,84]. Moreover, as shown above, cINs rarely exhibit proliferation markers indicative of recent division in the SVZ, and BrdU birth dating studies suggest their origin during embryonic development [31]. Taken together, the available data suggests that adult migration from the SVZ represents a minor or injury-specific pathway rather than the primary source of physiological layer II cINs. Newly generated DCX/PSA-NCAM neurons occasionally observed in the adult cortex likely represent a fundamentally distinct population responding exclusively to pathological insults, rather than a steady-state mechanism for replenishing the embryonic reservoir. Additionally, this injury-induced influx of migrating cells may explain the presence of proliferative marker-expressing cells among layer II cINs and inhibitory neurons, since the SVZ predominantly produces calretinin-expressing (CR+) GABAergic interneurons that migrate to the cortex, particularly post-stroke [102,103].

### 2.4. cIN Differentiation

The fate of cINs remained unclear for a long time—what happens to these cells after maturation and into which neuronal type do they differentiate? This question was illuminated through studies utilizing transgenic DCX-CreERT2/flox-EGFP mice, which enabled the tracking of DCX-positive neurons even after the cessation of marker expression [37,104]. Monitoring changes in the electrophysiological properties of cINs and the expression profile of their molecular markers at different stages of ontogeny in a transgenic mouse line revealed that cINs are capable of maturing and integrating into pre-existing neuronal networks of the mouse piriform cortex [37,79,105]. The cINs (complex cells) can awaken both in young and aged animals, and upon maturation they differ from neighboring principal neurons of the piriform cortex by exhibiting predominantly GABAergic input and generating action potentials at lower frequencies [79,105]. However, the nature of cIN differentiation remains a matter of debate. The majority of studies (Table 2) conducted on the paleocortices of rodents, including DCX-CreERT2/flox-EGFP mice, and in the neocortices of cats, guinea pigs, rabbits, and humans, indicate that cINs express typical markers of glutamatergic neurons (VGLUT1, CUX1, CTIP2, TBR1, and CAMKII) while lacking expression of markers associated with other neuronal types (GAD67, nNOS, NPY, CCK, SRIF, and VIP) [31,35,36,37,84]. Conversely, studies of cIN populations in the neocortices of guinea pigs, cats, and nonhuman primates demonstrate co-expression of GABAergic interneuron markers (GAD67 and GABA) and nitrergic interneuron markers (nNOS and NADPH-d), leading some authors to propose that cINs may differentiate into inhibitory neurons [46,65,66,86]. R. König and colleagues posit that these discrepancies might stem, in part, from inconsistent morphological criteria and the use of a single marker (DCX) for cIN detection [40]. They suggest that combining double immunolabeling for DCX and PSA-NCAM with additional detection of Tbr1, a highly specific marker for identifying pallium-derived immature glutamatergic neurons, could yield a more reliable classification of cINs. The possibility of both fates, and regional/species divergence, remains an open question requiring more methodologically harmonized fate-mapping studies.

## 3. Factors Modulating cINs Phenotype

### 3.1. Neurotransmitter Modulation and Pharmacological Effects

In the rat piriform cortex, dopaminergic signaling through D2 receptors (expressed on ~55% of PSA-NCAM+ cells) accelerates cIN maturation, as evidenced by opposite shifts in immature cell numbers following chronic D2 agonist (2-N-phenethyl-N-propyl amino-5-hydroxytetralin hydrochloride) or antagonist (haloperidol) administration [52]. Similarly, noradrenergic transmission exerts robust bidirectional control: norepinephrine depletion (in genetic models or by pharmacological agents) dramatically increases DCX+ and PSA-NCAM+ cell numbers, whereas enhancing noradrenergic signaling decreases these populations [87]. Furthermore, NMDA receptor antagonism increases immature marker expression, suggesting that basal glutamatergic signaling also suppresses these phenotypes [72]. This bidirectional control suggests that monoamines, including dopamine and noradrenaline, together with glutamate can serve as a maturation-promoting signal. Disruption of monoamines- or NMDA-receptor-mediated signaling may maintain or re-induce immature phenotypes in cortical neurons, indicating that these neurotransmitter systems play a critical role in regulating the transition from immature to mature states in cIN populations.

PSA depletion through endoneuraminidase-N treatment provides direct evidence for PSA-NCAM’s role in maintaining cIN immaturity. Targeted enzymatic removal of polysialic acid chains accelerates multiple aspects of maturation: increased NeuN expression frequency, enhanced axon initial segment development, and expanded dendritic arbors with increased spine density [73]. The mechanism involves removal of the anti-adhesive PSA coat, allowing formation of cell–cell contacts and synaptic connections that drive maturation programs [23,69]. These experiments demonstrate that PSA-NCAM presents more than a passive marker, but an actor actively maintaining cINs in an immature state through its insulating properties. The rapid maturation following PSA removal (within 2 weeks) [73] suggests that cINs are poised for differentiation and require only the removal of inhibitory signals.

While these pharmacological interventions provide strong evidence for the molecular regulation of cINs, it is important to note that most of these studies rely on systemic drug administration and static immunohistochemical snapshots [52,72,87]. The dynamic functional consequences of this induced maturation—for example, whether these cells successfully integrate into active cortical circuits or eventually undergo apoptosis—remain largely unexplored.

### 3.2. Stress-Related Factors

Chronic and early life stress disrupts neuroplasticity by altering the function, connectivity, and structure of key brain regions such as the hippocampus, amygdala, and prefrontal cortex, leading to long-lasting impairments in learning, memory, and emotional regulation [106]. Recent studies have demonstrated the impact of stress on the cIN population, with the potential to negatively affect their maturation, survival, and functional integration. The early-life stress model through maternal separation protocol in mice results in a reduced number of DCX/PSA-NCAM-positive immature neurons in the piriform cortex and impairs adaptive plastic potential of these cells in response to cognitive and sensory challenges [64,107]. While this review focuses on cortical populations, it is worth noting that similar stress-induced alterations of immature neurons have also been observed in the amygdala [78], suggesting a shared vulnerability of these delayed-maturing networks across different brain regions. This suggests that early adversity can alter the molecular program underlying cIN maturation, with possible long-term consequences on emotional and cognitive behaviors. Chronic stress and chronic corticosterone treatment produce differential effects on PSA-NCAM+ and DCX+ immature neurons in the piriform cortex. Chronic restraint stress (21 days) increases the number of PSA-NCAM+ and DCX+ cells in layer II of the adult rat piriform cortex, whereas chronic corticosterone administration decreases these populations [53]. This suggests that while stress activates plasticity mechanisms involving immature neurons, sustained elevation of glucocorticoids may suppress their expression or accelerate their differentiation. The opposing effects indicate that stress impacts immature neurons through both glucocorticoid-dependent and glucocorticoid-independent pathways. In another study, J. Nacher and his team demonstrated that in prefrontal cortical and limbic regions, chronic restraint stress (10 days) modulates PSA-NCAM expression and perineuronal nets, affecting interneuronal plasticity [108]. Specifically, PSA-NCAM expression increased in the CA1 area of the hippocampus (stratum lacunosum-moleculare), but no changes were found in the medial prefrontal cortex and basolateral amygdala [108]. This fact may indicate that non-newly formed cINs are more resistant to stress than immature cells of classical neurogenic niches.

### 3.3. Sensory Stimulation/Deprivation

M. A. Gomez-Climent and colleagues propose that olfactory bulbectomy induces the differentiation of cINs in the adult rat piriform cortex layer II [109]. Following unilateral olfactory bulbectomy, the number of PSA-NCAM+ and DCX+ cINs decreases in the ipsilateral piriform cortex, paralleled by a corresponding increase in the number of NeuN-expressing mature neurons [109]. In contrast to bulbectomy, odor-conditioned aversion paradigms did not significantly affect the population of cINs in the piriform cortex, suggesting that passive olfactory learning alone is insufficient to trigger their maturation [109]. Another study investigated how olfactory stimuli (sequential exposure to water, peanut butter, and rat bedding) affected cINs in the developing piriform cortex of CD1 mice at different postnatal ages (P2, P21, and P60) [54]. The researchers found that cINs responded to olfactory stimulation in an age-dependent manner. At P21, olfactory stimulation increased the number of non-proliferating DCX+/PSA-NCAM+ cells, while at P60 it mainly increased proliferating DCX+/PSA-NCAM+/Ki67+ neuroblasts [54]. These findings demonstrate that immature neurons in the piriform cortex layer II are responsive to activity-dependent signals from olfactory bulb inputs, and that disruption of this afferent input serves as a critical trigger for their differentiation and contributes to brain plasticity, particularly during the later stages of postnatal development.

### 3.4. Developmental and Age-Related Factors

cINs demonstrate profound changes across developmental stages. Most cINs are generated during embryonic development, particularly around embryonic day 15.5 in rodents [31]. In humans, during infancy and early childhood, DCX+ neurons are widely distributed throughout layer II of the cerebral cortex, but their quantity and distribution progressively narrow with advancing age, becoming largely restricted to the temporal cortex by old age [47]. It should be noted that a similar age-related maturation and depletion of immature neurons also occur in the paralaminar nucleus of the amygdala [27,78], though detailed discussion of this subcortical population falls outside the scope of this review. This developmental timing often coincides with major hormonal changes and emotional circuit development, suggesting coordinated regulation of immature neuron maturation with broader developmental programs. Similar results of age-related cell decline in different brain regions have also been demonstrated in mice, Chinese tree shrews, rabbits, sheep, and cats [28,33,37,66,110,111]. It is important to note that L. Bonfanti and colleagues demonstrated that the rate of age-related decline in cIN populations exhibits pronounced interspecies variation. While the pool of these cells is rapidly depleted in rodents as they mature, this decrease is substantially slower and delayed in larger-brained, gyrencephalic species, allowing them to maintain high numbers of cINs for prolonged periods [28,33].

The expression level of PSA-NCAM+ and DCX+ is modulated in response to a variety of factors, representing a complex interplay of neurotransmitter systems, enzymatic processes and environmental influences (Table 3). However, since only a limited number of studies investigated the differentiation and subsequent fate of cINs, for example, using transgenic reporter animal lines and electrophysiological recordings [37,79,104], it remains unclear whether the decrease in PSA-NCAM and DCX expression reflects neuronal maturation and, moreover, what mechanisms underlie the observed increase in PSA-NCAM and DCX expression (see Section 5 and Table 4).

## 4. Neurological Diseases

The emerging evidence linking cIN populations to various neurological pathologies, particularly epilepsy, traumatic brain injury and intoxication, suggests that these neurons may serve as critical nodes in the pathophysiology of brain disorders (Table 5).

Temporal lobe epilepsy (TLE) is the most common form of focal epilepsy in adults and is characterized by recurrent seizures originating in the temporal lobe, often presenting as complex partial seizures with features such as automatisms, altered consciousness, and auras [112,113,114]. Given that at least a subset of cINs differentiates into excitatory glutamatergic neurons [36,37,79], their potential involvement in the formation of aberrant networks underlying pathological hyperexcitability has been proposed [115,116]. Clinical studies investigating the impact of TLE on the cIN populations present conflicting findings, largely due to confounding methodological variables. Some reports demonstrate increased density and altered morphology of DCX+ cells in the temporal cortices of TLE patients [58,83]. For instance, a subset of these cells in mesial TLE patients co-expressed the proliferation marker PCNA and the early neuronal marker TuJ1, leading to the hypothesis that they might represent newly generated neurons migrating from the temporal ventricular horn [83,99]. However, these findings must be interpreted with caution. Comparisons between surgical TLE samples and postmortem controls are inherently biased by delayed fixation in postmortem tissues, which accelerates proteolytic degradation and reduces antigen detectability [117,118], alongside imprecise age matching [58]. Conversely, a study analyzing both human surgical samples and a pentylenetetrazol model of TLE in rats found no significant alterations in the cIN population across various cortical regions [36]. Nevertheless, it is crucial to emphasize that these negative results cannot be considered definitive due to the severe limitation of small sample sizes [36]. Similar inconsistencies are evident in developmental disorders such as focal cortical dysplasia type Ia. In this disorder, pronounced bands of DCX+ neurons have been observed in layer II of the affected children’s brains [59]. The increased expression of DCX shows an age-related decline, indicating either delayed cortical maturation or localized plasticity during critical developmental stages. However, these cells are almost undetectable in adult specimens or when using paraffin-embedding techniques [36,59]. This discrepancy highlights how tissue handling, specifically fixation protocols and the quality of antigen preservation, can significantly affect cIN quantification. Overall, while epilepsy likely affects cIN populations, at least in the temporal cortex, determining whether this is due to maladaptive plasticity (aberrant neurogenesis) or a non-specific response requires robust, standardized methodologies. There is also a critical gap regarding the human piriform cortex. Despite the central role of this region in seizure generation and the favorable outcomes of its resectioning in mesial TLE [119,120,121,122], and the extensive characterization of cINs in the rodent piriform cortex [72,107], the presence and phenotype of immature neurons in this human region remains largely unexplored.

Stroke is a cerebrovascular emergency defined by the acute disruption of cerebral blood flow, leading to rapid neuronal death and ranking as a major global contributor to mortality and lasting disability [123,124]. In the context of this pathology, preclinical models suggest a potential mobilization of immature neurons in response to ischemic injury. Specifically, in a rat model of focal cerebral ischemia, an increase in the number of DCX+ cells was observed in the ischemic penumbra of the corpus callosum and neocortex, particularly on days 5, 10 and 14 following stroke [90]. Since control animals had no detectable DCX+ cells in the cortex, the authors suggest that neuroblasts may appear either by migration from the SVZ or arise from local precursors [90]. Notably, no co-expression of DCX and the proliferation marker Ki-67 [125] was detected in the ischemic penumbra, indicating that DCX+ neurons in these regions are postmitotic [90].

Traumatic brain injury (TBI) is a major global cause of mortality and disability, characterized by an acute mechanical disruption of brain tissue followed by a complex cascade of secondary pathophysiological processes, including neuroinflammation and progressive neurodegeneration [126,127,128]. In a murine-controlled cortical impact model, moderate-to-severe TBI induced a pronounced reduction in PSA-NCAM expression at both the primary injury site and the contralateral cortex [101]. This reduction was hypothesized to reflect the injury-induced differentiation of immature neurons as a compensatory response [101]. Conversely, an analysis of perilesional cortical tissue from human TBI patients revealed a contradictory pattern: an up-regulation of the neural stem cell marker SOX2 [92,129] and immature neuronal markers, including DCX, PSA-NCAM, and TUC-4 [129]. Furthermore, unlike murine models and healthy human brains where proliferation markers in cINs are negligible, the majority of these DCX+ cells in human TBI samples co-expressed Ki-67 [92]. This injury-induced proliferative activity parallels observations in some TLE patients, where DCX/PCNA co-expression has been reported [83]. However, it contrasts sharply with ischemic stroke models, which demonstrate an increase in strictly postmitotic DCX+ neurons lacking Ki-67 [125].

## 5. Open Questions and Future Perspectives

Despite remarkable progress in characterizing cINs over the past two decades, fundamental questions regarding their biology, function, and therapeutic potential remain unanswered. This section synthesizes the major knowledge gaps identified throughout this review and proposes strategic directions for future investigation. We organize these open questions into Table 4.

The most fundamental unresolved question concerns the molecular programs maintaining cINs in an immature state for extended periods. While PSA-NCAM contributes through its anti-adhesive properties, enzymatic removal accelerates but does not immediately complete maturation [73], indicating that additional mechanisms are operative. Whether transcriptional repressor complexes, epigenetic modifications, or microRNA networks actively suppress maturation programs remains unknown. Complementary to maintenance mechanisms, the signals triggering cIN maturation require systematic characterization. Although dopaminergic, noradrenergic, and glutamatergic modulation influence cIN populations [52,72,87], the downstream molecular cascades and whether diverse triggers converge on common pathways remain largely uncharacterized. The terminological landscape surrounding these cells reflects historical confusion and methodological limitations. Various terms including “immature neurons,” “cortical layer II immature neurons”, “layer II doublecortin-expressing neurons”, “dormant precursors,” “non-proliferative neuronal precursors”, “tangled cells”, “complex cells”, “extraverted neurons”, “semilunar cells” and “arrested development” have been applied inconsistently across the literature [23,31,32,79]. The extent of heterogeneity within cIN populations, particularly whether functionally distinct subpopulations exist beyond maturational stages and whether regional differences (piriform cortex, neocortex, and amygdala) reflect fundamentally distinct populations, requires systematic investigation. The persistent controversy regarding excitatory versus inhibitory differentiation fates (see Section 2.4.) may reflect genuine heterogeneity demanding harmonized cross-species studies. The functional consequences of cIN circuit integration remain almost entirely unknown. Unlike hippocampal adult neurogenesis, where causal relationships with cognition are established, no study has demonstrated that cINs are necessary or sufficient for any specific function. Development of genetic tools enabling selective cIN manipulation, distinct from adult-born neurons, would be transformative. Such experiments could determine whether cINs contribute to olfactory processing, emotional regulation, or social cognition, as suggested by their anatomical distribution. The inverse phylogenetic relationship between cIN abundance and adult neurogenesis demands mechanistic explanation. Comparative studies across broader taxonomic ranges, including unstudied groups such as cetaceans, could illuminate these evolutionary patterns. Clinically, cIN alterations in epilepsy and traumatic brain injury raise questions requiring resolution. The potential contribution of cINs to epileptogenesis warrants attention given their location in piriform and temporal cortices, regions implicated in seizure generation [119,121,122]. Looking forward, the field must establish whether cINs represent viable therapeutic targets. Prerequisites for clinical translation include the demonstration of beneficial effects in disease models, identification of druggable targets, establishment of safety margins regarding aberrant plasticity risks, and validation of cross-species translation. The answers to these questions will advance fundamental understanding of brain plasticity while potentially revealing novel therapeutic opportunities for neurological conditions where harnessing endogenous cellular resources could complement current treatments.

## 6. “Neurogenesis Without Division”: Concluding Remarks

This review synthesizes current understanding of cINs—a distinctive neuronal population that challenges traditional paradigms of adult brain plasticity. Several key principles emerge from the accumulated evidence.

First, cINs represent a biologically distinct entity from adult-born neurons, characterized by prenatal generation followed by protracted maintenance in an immature state. The mechanisms underlying this arrested development involve PSA-NCAM-mediated reduction of cell–cell adhesion, effectively insulating these neurons from activity-dependent maturation signals, combined with regulatory control by monoaminergic and glutamatergic neurotransmitter systems. Their molecular signature, including DCX+, PSA-NCAM+, NeuN−/low with Tbr1 expression, provides a reliable, though not absolute, identification framework when markers are employed in combination.

Second, the inverse phylogenetic relationship between cIN abundance and canonical adult neurogenesis represents a fundamental organizing principle with profound implications. Rather than a definitive evolutionary adaptation, this inverse correlation may reflect distinct biophysical and allometric scaling differences across mammalian lineages. The mechanisms maintaining neural plasticity seem to diverge according to biological parameters such as brain size, metabolic demands, and lifespan. For translational research, it is important to be cautious when extrapolating findings from studies in rodents, which have robust adult neurogenesis and restricted cINs, to humans and other large-brained mammals, where the balance may be reversed.

Third, cINs demonstrate remarkable responsiveness to physiological and pathological stimuli. Dopaminergic and noradrenergic modulation, NMDA receptor signaling, stress exposure, sensory experience, and the aging process all influence cIN populations, suggesting their integration into broader networks of activity-dependent plasticity. The bidirectional nature of many regulatory influences—where the same system can promote either maintenance or maturation depending on context—indicates sophisticated regulatory logic that remains incompletely understood.

Fourth, the clinical relevance of cIN alterations in neurological conditions including temporal lobe epilepsy, traumatic brain injury, and stroke positions these cells as potential contributors to both maladaptive circuit reorganization and endogenous repair mechanisms. The observation that pathological conditions may induce proliferative activity in normally postmitotic cIN populations raises intriguing questions about their regenerative potential while simultaneously suggesting caution regarding their possible contribution to aberrant network excitability. Substantial gaps remain in our understanding of cINs biology. The precise molecular programs maintaining cellular immaturity, the triggers initiating differentiation, the functional consequences of cIN integration into existing circuits, and the behavioral or cognitive correlates of cIN-mediated plasticity all require systematic investigation. The persistent debate regarding cIN differentiation fate—whether they become excitatory glutamatergic neurons, inhibitory GABAergic interneurons, or both depending on context—reflects deeper uncertainties about regional and species-specific heterogeneity within this population.

Looking forward, methodological harmonization will be essential for resolving current controversies. Standardized tissue processing protocols, consistent application of marker combinations including DCX, PSA-NCAM, and Tbr1, and broader adoption of genetic lineage-tracing approaches should become field-wide priorities. The development of tools enabling selective manipulation of cINs in vivo will be crucial for determining their functional contributions to cognition, behavior, and disease pathophysiology. The therapeutic potential of cINs merits particular attention. As an endogenous population of cells poised for circuit integration, they represent attractive targets for regenerative medicine strategies. However, their potential involvement in pathological hyperexcitability demands careful risk–benefit analysis. Ultimately, harnessing cINs for brain repair will require a mechanistic understanding sufficient to promote their beneficial contributions while avoiding adverse consequences.

## Figures and Tables

**Table 1:** Comparison of cINs versus adult-born neurons from canonical neurogenic niches.

Characteristic	Cortical Immature Neurons	Adult-Born Neurons
Developmental Timing	Prenatally generated (embryonic); maintain immature phenotype into adulthood [22,28]	Continuously generated throughout adult life from SVZ/SGZ stem cells [29,30]
Proliferation Status	Mostly post-mitotic [31,32,33]	Initially proliferative; become post-mitotic upon differentiation [29,34]
Morphology—Immature	Two types: (1) Small tangled cells with short processes; (2) Complex cells with apical dendrites [35,36,37]	Stereotypical developmental progression from round cells to mature granule cell/interneuron morphology [38,39]
Morphology—Mature	Mostly develop into pyramidal-like neurons with typical dendritic arbor and spines [37,40]	Hippocampus: granule cells; OB: interneurons (granule cells; periglomerular cells) [38,41]
Location—Rodents	Restricted mainly to paleocortical layer II (piriform) [22,33,42]	SVZ → olfactory bulb; SGZ → dentate gyrus granule cell layer [22,43,44]
Location—Large Mammals	Widespread in neocortical layer II; amygdala; associative cortical areas [22,45,46]	Limited adult neurogenesis in SVZ/SGZ; controversial in primates/humans [22,44]
Species Distribution	Inverse correlation with adult neurogenesis—more abundant in large-brained mammals [22,44]	More prominent in small mammals; reduced in large-brained species [22,44]
Age-related Changes	Numbers decrease progressively with age as cells mature and differentiate [28,46,47]	Adult neurogenesis declines dramatically with age in most species [48,49]
Functional Role	Proposed structural plasticity reservoir [44];	Pattern separation (hippocampus); olfactory discrimination (OB); memory formation [50,51]
Regulation	Modulated by neurotransmitters; stress; sensory stimulation [52,53,54]	Regulated by neurogenesis factors; activity; stress; environmental enrichment [50,55,56,57]
Clinical Relevance	Altered in epilepsy; stress disorders; potential role in psychiatric conditions [36,58,59]	Implicated in depression; cognitive decline; neurodegenerative diseases [50,55,60,61]

OB—olfactory bulb.

**Table 3:** Factors modulating cINs.

Factor	Effect	Region	Species	Reference
Aging	Progressive ↓ PSA-NCAM+ and DCX+ cells	Neocortex/piriform cortex layer II; amygdala	Rat, cat, primate, human	[28,46,47]
Neurotransmitter modulation and pharmacological effects				
Dopamine D2 receptor antagonist (haloperidol)	↑ PSA-NCAM+ cells	Piriform cortex layer II	Rat	[52]
Dopamine D2 receptor agonist (PPHT)	↓ PSA-NCAM+ cells	Piriform cortex layer II	Rat	[52]
Norepinephrine depletion (DSP-4)	↑ PSA-NCAM+, DCX+ and nestin cells	Piriform cortex layer II	Rat	[87]
α2-adrenergic receptor agonist (guanabenz)	↑ PSA-NCAM+, DCX+ and nestin cells	Piriform cortex layer II	Rat	[87]
α2-adrenergic receptor antagonist (yohimbine)	↓ PSA-NCAM+, DCX+ and nestin cells	Piriform cortex layer II	Rat	[87]
PSA enzymatic depletion (Endo-N)	Accelerated maturation (loss of PSA-NCAM)	Piriform cortex layer II	Mouse	[73]
NMDA receptor antagonist (CGP43487)	↑ PSA-NCAM+ cells (7 days) and DCX+ cells (21 days)	Piriform cortex	Rat	[72]
Stress-related factors				
Chronic stress	↑ PSA-NCAM+ cells	Piriform cortex	Rat	[53,108]
Chronic corticosterone	↓ PSA-NCAM+ cells	Piriform cortex	Rat	[53]
Early life stress	↓↑ DCX+/PSA-NCAM+ cells	Piriform cortex; amygdala	Mouse	[64,78,107]
Sensory stimulation/deprivation				
Olfactory bulbectomy	↓ DCX+/PSA-NCAM+, ↓ GAD67 ↑ NeuN cells	Piriform cortex	Rat	[109]
Olfactory stimulation	↑ DCX + PSA-NCAM (21 day after birth (dab)) ↑ DCX/PSA-NCAM/Ki67+ (60 dab)	Piriform cortex	Mouse	[54]

**Table 4:** Selected open questions related to investigating cortical immature neurons.

Category	Question
Terminology	What standardized terminology should be adopted for cINs to avoid confusion with other DCX+/PSA-NCAM+ cell populations, and how can the field achieve consensus on definitional criteria?
Molecular mechanisms	What are the precise molecular programs—transcriptional, epigenetic, and post-translational—that maintain cINs in an immature developmental state for extended periods?
Molecular mechanisms	What are the specific signals that trigger the transition from quiescent immature state to active maturation in cINs, and do different triggers activate distinct differentiation programs?
Molecular mechanisms	What role do specific microRNAs play in maintaining the immature phenotype of cINs, and can their manipulation accelerate or prevent maturation?
Molecular mechanisms	Do cINs possess unique epigenetic signatures (DNA methylation or histone modifications) that distinguish them from both immature adult-born neurons and mature cortical neurons?
Molecular mechanisms	What prevents cINs from undergoing apoptosis during their extended period of incomplete differentiation, and do they express unique anti-apoptotic programs?
Heterogeneity	What is the extent of heterogeneity within cIN populations regarding origin, molecular profile, functional capacity, and regulatory mechanisms across brain regions?
Distribution	Are there undiscovered populations of non-newly generated DCX+/PSA-NCAM+ immature neurons in brain regions beyond the studied regions?
Human translation	How can findings from rodent cIN studies, primarily in paleocortical regions, be translated in order to understand neocortical cINs in humans, where they are more prevalent?
Development	Are cINs specified as a distinct population during embryonic development or are they formed from a common pool of precursors?
Development	Which embryonic signaling gradients or transcription factor combinations determine whether a neuron generated during cortical neurogenesis becomes a cIN versus a conventionally maturing neuron?
Origin	What is the definitive contribution of SVZ-derived migration, as opposed to local persistence, to adult cIN populations under normal physiological conditions?
Origin	Are the proliferating DCX+ cells observed under pathological conditions (TBI and TLE) derived from resident cINs, local progenitors, or migrating SVZ neuroblasts?
Electrophysiology	Do cINs contribute to network activity through subthreshold membrane oscillations or gap junction coupling before acquiring action potential generation capacity?
Electrophysiology	What is the critical period during which newly matured cINs exhibit enhanced synaptic plasticity compared to existing circuit elements?
Circuit integration	How do newly matured cINs establish appropriate synaptic partnerships—which molecular cues guide their axonal targeting and dendritic positioning?
Circuit integration	What proportion of cINs that initiate maturation successfully integrate into functional circuits versus undergoing elimination through activity-dependent pruning?
Function	Which specific cognitive, sensory, or behavioral functions depend on the presence and maturation of cINs?
Function	Do cINs serve as a cellular “reserve” that can be recruited during learning, environmental enrichment, injury, or disease, and what determines their mobilization?
Function	Do cINs in the piriform cortex contribute to olfactory learning and memory, and if so, at which stage of processing—pattern separation, pattern completion, or memory consolidation?
Function	Does the rate of cIN maturation correlate with individual differences in learning capacity, cognitive flexibility, or stress resilience?
Function	Can sensory experience or cognitive training selectively recruit cIN maturation in relevant cortical regions?
Disease	How does neuroinflammation affect cIN maintenance, maturation, and survival—do microglia regulate cIN phenotype?
Disease	Can aberrant cIN maturation contribute to the development of cortical malformations or heterotopias when developmental regulation fails?
Disease	Can cINs contribute to epileptogenesis through integration into hyperexcitable networks, or do they provide compensatory inhibition that limits seizure propagation?
Disease	Do cINs in the human piriform cortex play a specific role in temporal lobe epilepsy given this region’s proposed function as a seizure amplification zone?
Disease	Do cIN alterations precede symptom onset in neurodevelopmental disorders, potentially serving as early biomarkers or therapeutic targets?
Disease	What is the fate of cINs in neurodegenerative diseases—are they selectively vulnerable, relatively resistant, or capable of compensatory responses?
Sex Differences	Do cIN populations differ between males and females, and are they regulated by sex hormones during puberty, reproductive cycling, or menopause?
Hormonal	How do thyroid hormones, which profoundly influence brain development and maturation, affect cIN maintenance and differentiation?
Aging	Does the rate of cIN decline correlate with age-related cognitive decline, and can interventions that preserve cIN populations maintain cognitive function?
Aging	Are there “long-lived” cINs that persist throughout the lifespan versus cINs that mature during specific developmental windows?
Aging	How does aging affect the capacity of remaining cINs to undergo maturation in response to appropriate stimuli?
Comparative	Do marine mammals (cetaceans and pinnipeds) possess cIN populations, and if so, how do they compare to terrestrial mammals with similar brain sizes?
Comparative	Are there systematic differences in cIN properties between cortical regions with different evolutionary ages (paleocortex versus neocortex) within the same species?
Methods	Can single-cell multi-omics approaches (transcriptomics, epigenomics, and proteomics) identify molecular signatures unique to cINs that enable development of specific genetic tools?
Methods	What non-invasive imaging approaches might enable detection or monitoring of cIN populations in living humans?
Methods	How can the field establish standardized protocols for cIN quantification that account for tissue processing variables and enable cross-study comparisons?
Methods	Can organoid or assembloid models recapitulate cIN biology sufficiently to enable mechanistic studies not feasible in vivo?
Therapeutics	Can pharmacological modulation of PSA-NCAM (through polysialyltransferase inhibitors or endoneuraminidase-N delivery) be developed as a therapeutic strategy to promote cIN maturation?
Therapeutics	What is the therapeutic window for promoting cIN maturation after brain injury and does early versus delayed intervention produce different outcomes?
Therapeutics	Could cINs serve as targets for cell replacement strategies, either through transplantation of exogenous immature neurons or in situ reprogramming?
Therapeutics	Which biomarkers could serve as endpoints for cIN-targeted therapies in clinical trials?
Microenvironment	How does the composition of the extracellular matrix surrounding cINs differ from that surrounding mature neurons, and does it contribute to phenotype maintenance?
Microenvironment	How does vascular proximity influence cIN distribution, maintenance, and maturation, given known relationships between angiogenesis and neurogenesis?
Systems	Do cIN populations in different cortical regions coordinate their maturation timing, potentially through long-range circuit activity or hormonal signals?

**Table 5:** Alterations of cINs populations in neurological diseases.

Factor	Effect	Region	Species	Reference
Chronic lead exposure	↓ DCX+ cells	Neocortex layer II	Guinea pig	[91]
Small-vessel stroke	↑ DCX+ cells No DCX+/ki67 cells detected	Neocortex and corpus callosum	Rat	[90]
Traumatic brain injury	↑↓ DCX+/PSA-NCAM+, ↑ DCX+/ki67 cells	Neocortex	Mouse, human	[92,101]
Bipolar disorder	No changes	Neocortex	Human	[36]
Schizophrenia	No changes	Neocortex	Human	[36]
Major depression	No changes	Neocortex	Human	[36]
Epilepsy	No changes [36] ↑ DCX+ cells [58] ↑ DCX+/PCNA cells [58]	Neocortex (significantly in temporal lobe); piriform cortex	Human, rat	[36,58]
Focal cortical dysplasia type Ia	↑ DCX+ cells	Neocortex layer II	Human	[59]
Focal cortical dysplasia type IIb	No changes	Neocortex	Human	[59]
Grey matter heterotopia	No changes	Neocortex	Human	[59]
Pediatric hippocampal sclerosis	No changes	Neocortex	Human	[59]
Temporal lobe sclerosis	No changes	Neocortex	Human	[59]
Glioneuronal tumors	No changes	Neocortex	Human	[59]

## Data Availability

No new data were created or analyzed in this study. Data sharing is not applicable to this article.
